# Evaluating Serum Markers for Hormone Receptor-Negative Breast Cancer

**DOI:** 10.1371/journal.pone.0142911

**Published:** 2015-11-13

**Authors:** Michèl Schummer, Jason Thorpe, Maria Giraldez, Lindsay Bergan, Muneesh Tewari, Nicole Urban

**Affiliations:** 1 Fred Hutchinson Cancer Research Center, 1100 Fairview Ave N, Seattle, Washington, United States of America; 2 Department of Internal Medicine, Division of Hematology/Oncology, University of Michigan, Ann Arbor, Michigan, United States of America; 3 Divisions of Hematology/Oncology and Molecular Medicine and Genetics, Department of Internal Medicine, University of Michigan, Ann Arbor, Michigan, United States of America; 4 Department of Biomedical Engineering, University of Michigan, Ann Arbor, Michigan, United States of America; 5 Biointerfaces Institute, University of Michigan, Ann Arbor, Michigan, United States of America; 6 Center for Computational Medicine and Bioinformatics, University of Michigan, Ann Arbor, Michigan, United States of America; Mathematical Institute, HUNGARY

## Abstract

**Introduction:**

Breast cancer is the most frequently diagnosed cancer and the leading cause of cancer death in females worldwide. Death rates have been declining, largely as a result of early detection through mammography and improved treatment, but mammographic screening is controversial because of over-diagnosis of breast disease that might not require treatment, and under-diagnosis of cancer in women with dense breasts. Breast cancer screening could be improved by pairing mammography with a tumor circulating marker, of which there are currently none. Given genomic similarities between the basal breast cancer subtype and serous ovarian cancer, and given our success in identifying circulating markers for ovarian cancer, we investigated the performance in hormone receptor-negative breast cancer detection of both previously identified ovarian serum markers and circulating markers associated with transcripts that were differentially expressed in breast cancer tissue compared to healthy breast tissue from reduction mammaplasties.

**Methods:**

We evaluated a total of 15 analytes (13 proteins, 1 miRNA, 1 autoantibody) in sera drawn at or before breast cancer surgery from 43 breast cancer cases (28 triple-negative—TN—and 15 hormone receptor-negative—HRN—/ HER2-positive) and 87 matched controls.

**Results:**

In the analysis of our whole cohort of breast cancer cases, autoantibodies to TP53 performed significantly better than the other selected 14 analytes showing 25.6% and 34.9% sensitivity at 95% and 90% specificity respectively with AUC: 0.7 (p<0.001). The subset of 28 TN cancers showed very similar results. We observed no correlation between anti-TP53 and the 14 other markers; however, anti-TP53 expression correlated with Body-Mass-Index. It did not correlate with tumor size, positive lymph nodes, tumor stage, the presence of metastases or recurrence.

**Conclusion:**

None of the 13 serum proteins nor miRNA 135b identified women with HRN or TN breast cancer. TP53 autoantibodies identified women with HRN breast cancer and may have potential for early detection, confirming earlier reports. TP53 autoantibodies are long lasting in serum but may be affected by storage duration. Autoantibodies to TP53 might correlate with Body-Mass-Index.

## Introduction

Breast cancer is the most frequently diagnosed cancer and the leading cause of cancer death in females worldwide. In 2012 female breast cancer accounted for 25% (1.67 million) of the total new female cancer cases and 15% (521,900) of the total female cancer deaths [1]. Breast cancer death rates have been decreasing in North America and several European countries over the past 25 years, largely as a result of early detection through mammography and improved treatment [2].

Mammography is the standard breast cancer screening tool, which has led to the discovery of cancers in their early stages, pushing 5-year survival to 90% as opposed to 20% when the disease has already spread to distant organs [3]. However, its utility as a screening method is controversial [4, 5], due in part to an increased diagnosis of *in situ* breast disease [6], a condition that may or may not progress to invasive cancer. Furthermore, mammographic screening is less sensitive in women with dense breasts, a feature commonly found in younger women [7, 8]. Screening output could potentially be improved by pairing mammography with a tumor circulating marker. Currently, no markers for breast cancer screening or detection are in clinical use, but a few markers, e.g. carcinoembryonic antigen (CEA), or carbohydrate antigens 15–3 and 27.29, are being used and seem helpful for making decisions in the metastatic [9] or surveillance [10] setting. Many studies have focused on analyzing transcript markers in breast cancer, starting with Perou’s molecular portraits [11] and followed by numerous others. This has culminated in clinical tests for cancer outcome, such as Oncotype DX [12] and Mammaprint [13]. Few studies have catalogued protein expression in breast cancer [14, 15]. These did not provide information in healthy breast tissue, which prevent the markers’ use as diagnostic biomarkers. In 2011, autoantibodies to several glycopeptides of MUC1, the protein recognized by the cancer antigens CA15-3 and CA27.29, were found to discriminate sera drawn from breast cancer patients at diagnosis from benign and healthy controls [16]. However, when the same group tested autoantibodies to MUC1 in a nested case-control study involving over 1000 serum samples from women who later developed breast cancer and over 1300 matched controls, no differences were observed [17]. Another promising breast marker is autoantibodies to TP53 [18–20], found to be present in sera that were collected on average more than 150 days before a breast cancer diagnosis, although with a relatively small sample size [21].

Finding and validating new biomarkers is a difficult task [22]. We previously discovered Human epididymis protein 4 (HE4, WFDC2) as a marker for ovarian cancer, first as a transcript in tissue and, four years later, as protein in serum [23, 24], and identified several other genes and serum markers that similarly discriminated ovarian cancer from controls [25]. Given the genomic similarities between the aggressive basal breast cancer subtype and serous ovarian cancer [26, 27] and the hypothesis that biomarkers may overlap between these two subtypes [27], we investigated whether any of the serum markers that signal the presence of ovarian cancer could also signal presence of HRN or TN breast cancer.

Additionally, applying the successful ovarian marker discovery strategy, transcripts that we found to be differentially expressed in breast cancer tissue compared to healthy breast tissue from reduction mammaplasties [28] were also tested for their discriminatory potential as proteins in serum.

Next to proteins and autoantibodies, miRNAs are being studied as circulating markers. Micro-RNA-135b has been shown to be over-expressed in TNBC [29–31] and was chosen as a further candidate.

We tested women diagnosed with basal-like breast cancers because they exhibit genomic similarity to serous ovarian cancer [26] and have poor prognosis, potentially greatly benefitting from a serum marker. For reasons of availability of clinical data, we chose patients whose cancers were negative for estrogen, progesterone and HER2 receptor (triple-negative or TN), being aware that not all TN breast cancers have basal-like histology [32]. Independently, survival data for TN cancers are among the worst [33, 34]. Our population was augmented with another poor-prognosis sub-group, hormone receptor-negative (HRN) breast cancers positive for HER2 [35, 36].

We tested a total of 15 analytes in sera drawn at or before breast cancer diagnosis from 43 HRN cases (including a subset of 28 TN cases) and 87 matched controls. As reported below, we found that autoantibodies to TP53 discriminated HRN breast cancer cases from screening controls.

## Methods

### Study Population

Cases included 43 women with HRN breast cancer, enrolled prior to therapeutic surgery (lumpectomy or mastectomy) for invasive cancer at Swedish Medical Center (SMC) in Seattle, WA, USA. All cancers had ductal histology, 28 were TN and 15 were HRN and HER2-positive. Four patients received neo-adjuvant chemotherapy. Blood was drawn prior to surgery in all cases, either on the day of surgery (*n* = 12) or up to one week (*n* = 16), two to six weeks (*n* = 10) or seven to 30 weeks (*n* = 5) before surgery. For 21 of the 43 cases, a post-treatment blood draw was also obtained. To the 43 cases, 87 healthy controls were matched by age, time period of blood draw and ethnicity (**Table 1** and **S1 Table**). Controls were participating in mammography screening at SMC and had no history of cancer per SMC or the local Cancer Surveillance System [37], and did not develop cancer within four years. Blood draws for cases and controls followed the same protocol implemented by the same staff at Fred Hutchinson Cancer Research Center (FHCRC). For cases and controls, questionnaire data were available. Patient characteristics are summarized in **S1 Table**.

**Table 1:** Matching Cases to Controls.

Variable	Value	Invasive Cases	Healthy Controls	p-Value (Invasive vs. Healthy)
**N**	Sample Size	43 (33.1%)	87 (66.9%)	
**Ethnicity**	Asian-Filipino	1 (2.3%)	0 (0.0%)	0.42
	Black	0 (0.0%)	2 (2.3%)	
	Caucasian	31 (72.1%)	71 (81.6%)	
	Hispanic	1 (2.3%)	3 (3.4%)	
	Mixed	3 (7.0%)	5 (5.7%)	
	Unknown	7 (16.3%)	6 (6.9%)	
**Collection Date**	mean (sd)	2006-01-30 (317 days)	2005-11-29 (406 days)	0.34
**Date Range**	Oldest Collection	2004-04-23	2004-05-12	
	Newest Collection	2008-06-13	2008-06-06	
**Age at Blood Draw**	mean (sd)	56 (12)	54 (10)	0.55

### Four criteria for selection of 15 analytes (Table 2)

The goal of our research is the identification of markers or analytes for the detection of breast cancer. Expression of an analyte in cancer must therefore be known in relation to it its expression in a non-cancerous state (e.g. healthy blood or healthy breast tissue). This is reflected in the following four selection criteria. **Criterion 1 (ovarian cancer markers)**: Five protein analytes were chosen based on their performance as circulating markers in identifying high-grade serous ovarian cancer [25] and evidence for their role in breast or other cancers: **GDF15** (MIC-1) protein elevation in human serum is associated with oral squamous cell carcinoma [38], pancreatic cancer [39] and colon cancer [40]; **PKM** (pyruvate kinase muscle) protein was suggested a marker of clinical breast cancer disease [41] and is expressed in advanced breast cancers [42]; **SPARC** (osteonectin) protein is expressed in breast cancer tissue with potential prognostic value [43]; **CA125** (MUC16 protein) is associated with poor breast cancer prognosis [44] and is able to discriminate ER-negative breast cancer patients from controls with unknown ER status [45]; and **WFDC2** (HE4) protein, which we and others [46] have found to have low expression in benign breast disease and its expression in breast cancer tissue may be associated with worse survival [47]. **Criterion 2 (breast tissue transcript markers)**: Five protein analytes were selected based on their performance in our transcript-based breast cancer work comparing breast cancer tissue to that of normal tissue from breast reduction surgeries [28]: **COL1A1** (collagen, type 1, alpha 1) and **FN1** (fibronectin 1) showed higher mRNA expression in breast cancer tissues than in controls, **CTGF** (connective tissue growth factor) was less highly expressed in aggressive cancers vs. healthy controls, and **S100A7** (psoriasin) and **SPP1** (osteopontin) expression was associated with poor-outcome cancer [28]. All five transcripts were previously associated with breast cancer [11, 48–59] and the corresponding proteins are all expressed in breast cancer tissue [60]. Furthermore, CTGF protein expression in cancer tissue is associated with poor prognosis in some cancer types [61], S100A7 protein is expressed in breast cancer [62] and SPP1 protein is implicated in breast tumor progression [63] and significantly elevated in patients with TN breast cancer [64]. CTGF protein is present in blood of liver cancer patients [65] and S100A7 protein is found reduced in serum of psoriasin patients [66], FN1 is expressed in plasma of breast cancer patients [67] and higher in breast cancer serum than controls [68], SPP1 protein is present in serum [69] and elevated in that of breast cancer patients [70], SPP1 however failed to discriminate breast cancer cases from healthy controls in pre-diagnostic serum samples [71]. **Criterion 3 (poor outcome breast cancer)**: Three analytes, **CCL5** (RANTES) protein, **hsa-miR-135b** (microRNA 135b) and **Anti-TP53** (autoantibodies to p53) were chosen because of their association with poor breast cancer outcome: CCL5 was reported to be over-expressed in HRN breast cancer and to increase with cancer stage [72, 73], miR-135b is over-expressed in basal-like breast cancers and associated with estrogen receptor negativity and with poor survival and early metastasis [29–31]. Anti-TP53 was shown to be expressed in TN cancers [74]. CCL5 and autoantibodies to TP53 were also reported in breast cancer serum [21, 73]. **Criterion 4 (low expression in breast cancer)**: Lastly, two protein analytes were included which showed decreased protein expression in breast cancer tissue compared to healthy control tissue, **HOXA5** (homeobox A5) [75] and **SFRP1** (secreted frizzled-related protein 1) [76].

**Table 2:** Selection of 15 analytes and their sensitivity/specificity as serum markers.

	Selection Criterion	Sensitivity at 100% specificity	Sensitivity at 95% specificity	Sensitivity at 90% specificity
**CA125**	1	2.3%	11.6%	14.0%
**GDF15**	1	4.7%	11.6%	14.0%
**PKM**	1	0.0%	0.0%	17.5%
**SPARC**	1	2.3%	7.0%	14.0%
**WFDC2**	1	4.7%	9.3%	11.6%
**COL1A1**	2	0.0%	2.3%	2.3%
**CTGF**	2	7.0%	7.0%	9.3%
**FN1**	2	0.0%	11.6%	18.6%
**S100A7**	2	0.0%	2.3%	16.3%
**SPP1**	2	10.0%	10.0%	15.0%
**CCL5**	3	2.4%	12.2%	24.4%
**miR-135b**	3	0.0%	0.0%	4.7%
**Anti-TP53**	3	4.7%	25.6%	34.9%
**HOXA5**	4	2.3%	11.6%	11.6%
**SFRP1**	4	0.0%	7.0%	11.6%

Data from 43 cases and 87 controls. Marker selection criteria: (1) serum marker potential in high-grade serous ovarian cancer and involvement in breast cancer; (2) transcript involved in breast cancer; (3) association with poor breast cancer outcome; (4) protein decreased in breast cancer tissue compared to normal breast.

### Assays

Enzyme-linked immunosorbent assays (ELISA) were run in two formats, a conventional 96-well plate format (P) or on beads (B) on the Bio-Plex immunoassay platform (LifeSciences, Hercules, CA, USA). Assays were purchased when commercially available or developed from antibodies that were obtained from commercial sources or from collaborators. ELISA values were normalized by the mean of four samples of normal human serum (a pool of serum from 73 healthy female donors) present on each 96-well plate. Expression of miR-135b and a spiked-in control miRNA (cel-miR-39) was analyzed using individual Taqman RT-qPCR assays (Life Technologies, Foster City, CA), qPCR reactions were run on Viia7 Real-Time PCR System (Life Technologies, Foster City, CA) in duplicate using no-template controls. Cycle threshold (Ct) values were normalized as previously described [77]. Following is a list of the assays, if commercial, or the antibodies, if in-house (first: capture, second: detection). **CCL5** (B): Bio-Plex Pro Hu Cytokine Croup I RANTES (LifeSciences, Cat No 171G5022M). **COL1A1** (P): Rabbit pAb to Collagen I (Abcam, Cat No ab34710-100, 1 μg/ml), Col1A1 goat polyclonal IgG (Santa Cruz, Cat No SC8783, 1 μg/ml). **CTGF** (P): Anti-human CTGF Ab (Antigenix America, Cat No RHF461CK, 5 μg/ml), Anti-human CTGF Biotin Tracer (Antigenix America, Cat No FHF461CK, 0.5 μg/ml). **FN1** (P): Human Fibronectin Platinum ELISA (eBioscience, Cat No BMS2028). **GDF15** (P): A10.3 (diaDexus, 20 μg/ml), Biotinylated B2.2 (diaDexus, 1 μg/ml). **HOXA5** (P): 171–270 mAb (Abnova, Cat No H00003202-M06, 0.1 μg/ml), H-125 rabbit polyclonal IgG (Santa Cruz, Cat No SC28599, 2 μg/ml). **PKM** (P): Tumor M2-PK EDTA Plasma (ScheBo BioTech Inc, Cat No 8). **S100A7** (P): Ab817 (Kornelia Polyak, 1 μg/ml), Biotinylated Ab1068 (Kornelia Polyak, 1 μg/ml). **SFRP1** (P): H-90 (Santa Cruz, Cat No SC13939, 1 μg/ml), Human sFRP-1 Affinity Purified Polyclonal Ab, Goat IgG (R&D Systems, Cat No AF1384, 0.75 μg/ml). **SPARC** (P): Human SPARC Physcoerythrin mAb Clone 122511 (R&D Systems, Cat No IC941P, 1 μg/ml), SPARC [N50] Antibody (GeneTex Inc, Cat No GTX19528, 0.5 μg/ml). **SPP1** (B): Human Osteopontin Quantikine ELISA kit (R&D Systems, Cat No DOST00). **WFDC2** (B): mAb#172 (Brad Nelson, 1 μg/ml), Biotinylated mAb#144 (Brad Nelson, 1 μg/ml). **CA125** (B): RDI-TRK4C29-X306, RDI-TRK4C29-X52 (Research Diagnostics)[78]. **Anti-TP53** (B): p53-GST Recombinant Protein matching wild type and several isoforms, expressed in E. coli (Millipore, Cat No 14–865, 20 μg/ml) was coupled to magnetic beads (Life Sciences, Cat No MC10026-01) as described earlier [78] and serum was tested as described in the Supplement. **miR-135b** (rt-qPCR): For combined analysis with the ELISA values, Ct values were linearized using the formula *e* ^(40-Ct)^ where "*e*" is the base of the natural logarithm. Raw data (normalized) are summarized in **S2 Table**.

### Statistical analysis

Fisher's exact test was used to test for differences in distribution of ethnicity between cases and controls, and Student's t-test was used to test for differences in collection date and age at blood draw. Sensitivity and specificity of each analyte were evaluated using the receiver operating characteristic (ROC) curve analysis and the area under the ROC curve (AUC) was tested (H0: AUC = 0.5: no difference between cases and controls) using the Mann-Whitney test. Kendall's tau statistic was used to test for correlations between covariates and individual markers. Combination markers were calculated by fitting a logistic regression model with case status as the dependent variable and individual analyte as the independent variables, and were defined as the linear combination of the analyte values using the coefficients estimated by the regression model. A bootstrap analysis with 10,000 iterations was conducted to determine if the combination marker demonstrated a better than random improvement over the best marker alone. This analysis was performed using data from 40 cases and 86 controls because the analyte SPP1 had missing values for three cases and one control. Backward stepwise model selection was done using the Akaike's Information Criterion (AIC) via the 'stepAIC' procedure [79]. All statistical analyses were conducted in the R software package [80].

## Results

### Analyte Expression

**Fig 1:**
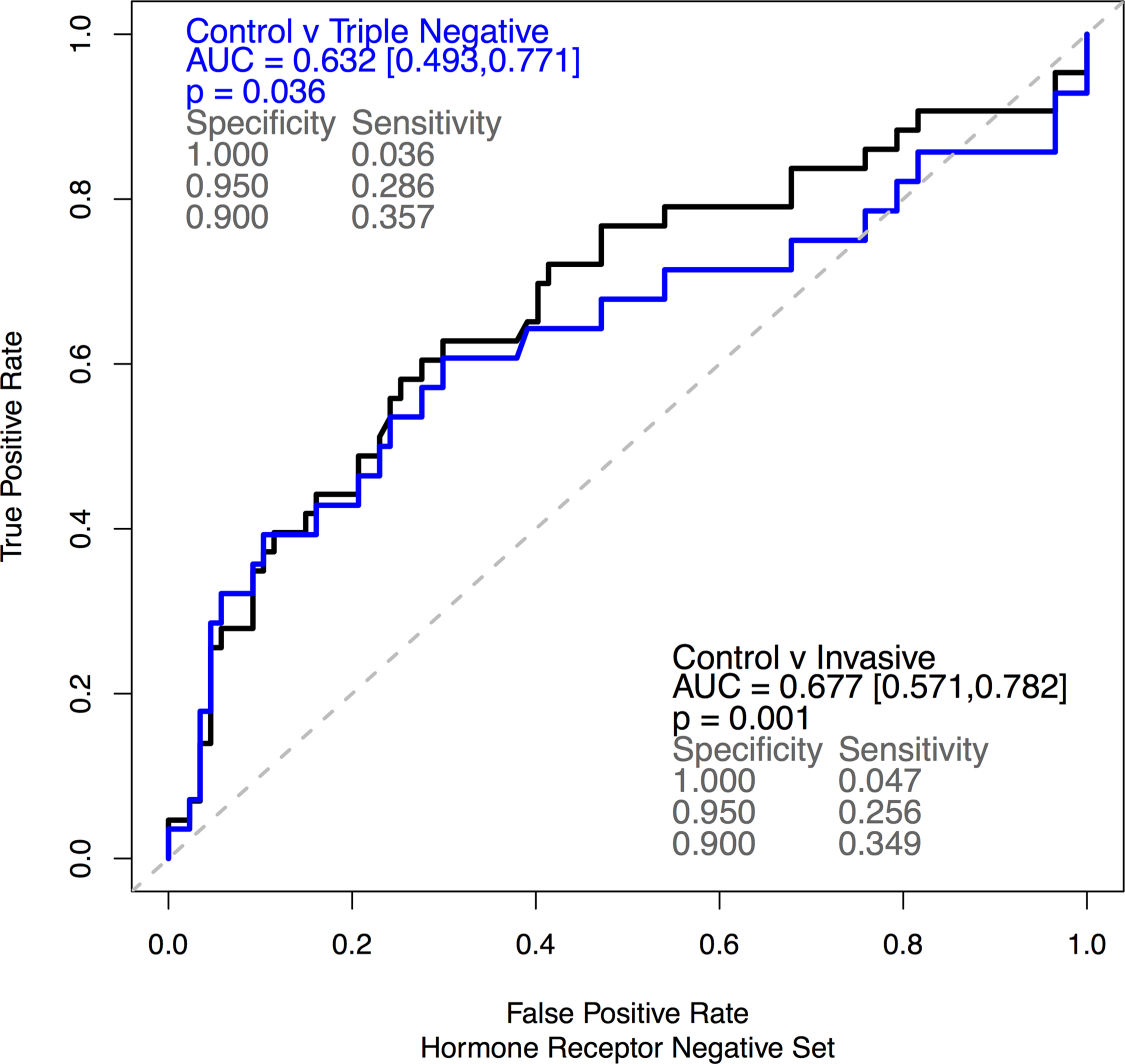
Autoantibodies to TP53 discriminate breast cancer sera from control sera. ROC curves showing the 43 HRN cases (black) and the 28 TN subset (blue) vs. 87 matched healthy controls.

All 15 assays were run on pre-surgical sera from 43 HRN breast cancer patients and 87 controls (**Table 1**). Analysis was performed on the full set of 43 HRN cases and on the subset of 28 TN cases. Comparing analyte sensitivities at 100%, 95% and 90% specificity ranked anti-TP53 ahead of all other 14 analytes (AUC: 0.677, p = 0.001, **Fig 1** and **Table 2**). ROC curves for the full HRN and the TN set are shown in **S1 Fig** and the corresponding dot plots are shown in **S2 Fig.** Autoantibodies to TP53 also outperformed the other markers in the TN subset, showing sensitivity values very similar to the ones obtained in the full set of cases (**Fig 1**). Conversely, some of the other 14 analytes performed slightly better in the TN subset than in the full set of breast cancer cases (**S2 Table**).

To test if any of the other 14 analytes may contribute to the sensitivity of anti-TP53, we selected two complementary analyte sets: the first set was chosen by hand using the sensitivities of individual analytes. It combines anti-TP53 with SPP1, FN1, CCL5 and GDF15. The second set was chosen through backward stepwise model selection using AIC, which resulted in a combination of anti-TP53 with FN1 and CTGF. Neither panel was found to have a better than random increase in either area under the curve (AUC) or the sensitivity of the panel at 90% or 95% specificity (p>0.094 for all comparisons, unadjusted for multiple comparisons, see **S4 Table**). The TN subset resulted in similar numbers (**S5 Table**).

Thus, of the 15-analyte panel only anti-TP53 warranted further analysis. Its relatively good performance was not influenced by the single outlier measurement in patient 5, which had a relative Mean Fluorescence Intensity (MFI) value of 22.45 compared to a mean of 0.78 (sd = 0.59) in the remaining 42 patients (**S2 Fig**). Removal of this serum sample marginally reduced anti-TP53 sensitivity at 95% and 90% specificity from 25.6% to 23.8% and from 34.9% to 34.5% respectively.

### Anti-TP53 levels throughout treatment

To test whether TP53 autoantibody levels remain steady throughout treatment, we compared anti-TP53 values of the pre-surgical samples to those in post-therapeutic samples which were available from 21 of the 43 cases. Post-therapy values dropped to an average of 81% of the diagnostic values. However, the same reduction in relative MFI values was observed in paired samples from 10 control individuals whose draws dated from the same time period and thus had similar blood draw intervals as the 21 cases (**S6 Table**). After adjustment for the date of serum collection, diagnostic and post-therapy values (as well as the values of the two control draws) were significantly correlated (**S3 Fig**).

### Anti-TP53 and confounding factors

To test whether anti-TP53 levels correlated with patient- and disease-related characteristics, we used Kendall's Correlation Test (**S7 Table**). Of the 22 covariates, body-mass-index (BMI) was the only with a significant correlation to anti-TP53 levels (p = 0.05, unadjusted for multiple comparisons), though the observed correlation was weak (0.317, **S4 Fig**) and was not significant when adjusted for multiple comparisons (p = 1, Bonferroni correction). Unfortunately, BMI data were only available in the cancer patients, and hence, statistical tests adjusted for BMI, which could eliminate the possibility that the correlation between anti-TP53 and case status were attributable to systematic differences in BMI, were not possible.

Tumor size, which is often correlated with analytes secreted by tumors, did not correlate with anti-TP53 and neither did the severity of the disease (positive lymph nodes, tumor stage, the presence of metastases and recurrence).

## Discussion

To investigate their potential as early detection markers, we measured the expression of 15 analytes in serum that was drawn prior to surgery in 43 cases with HRN breast cancer (and a 28-case TN subset thereof) and 87 matched healthy controls. Five of the evaluated analytes were proteins with marker potential in high-grade serous ovarian cancer (HGSOC), but this potential could not be replicated in this subset of breast cancer cases that is thought to be molecularly related to ovarian cancer [26].

Likewise, none of the analytes previously showing transcript-level over-expression in breast cancer cases gave a sufficient signal in serum, indicating that transcript expression in breast tissue is a poor indicator for a successful protein marker expressed in blood of HRN or TN breast cancer patients.

Given that years of research have not resulted in a protein marker for detection of breast cancer, and given that none of the protein analytes tested in this study and a recent study with over 800 cases and controls [45] display a significant signal at diagnosis, it may very well be that circulating proteins make poor breast markers.

Similarly, our selected microRNA, miR-135b, lacked adequate signal in serum for the diagnosis of HRN breast cancer.

Our best performing marker is indeed the body’s own antibodies to TP53 (anti-TP53), the expression of which has been associated with breast cancer before. Incidentally, anti-TP53 has been shown to have detection marker potential in HGSOC [81] and preliminary results by our group in a set of 728 sera (123 ovarian cancer cases, 369 healthy controls, 144 benign ovarian disease, 92 surgical controls) showed performance similar to that reported here in breast cancer (unpublished).

In 1982 Crawford *et al*. reported the presence of autoantibodies to TP53 in sera of women with breast cancer [82], and since then, multiple surveys have found anti-TP53 in 9–34% of women with breast cancer [18–20]. Comparing cases to controls, in 2012 Lu *et al*. reported that anti-TP53 had 35% sensitivity at 90% specificity in detecting breast cancer. Most importantly though, they also found 6% of pre-diagnostic breast cancer samples to be positive for this analyte [21], hinting at its potential for early diagnosis. Our data confirm these results and further suggest that anti-TP53 is potentially useful for the detection of poor-outcome breast cancers.

Our data further show that TP53 autoantibody levels are independent of tumor size, cancer stage, the presence of metastases or the presence of positive lymph nodes, again suggesting a potential to detect breast cancer in its early stages. Furthermore, diagnostic and post-therapeutic samples from the same patient show similar anti-TP53 expression after adjustment for time in the freezer. Due to the relatively small sample size and the wide confidence interval for the trend line for time in the freezer, the latter statement must be interpreted carefully, in particular because some autoantibody levels decrease with increased storage time [83] rather than increase as we observed for anti-TP53.

Given the relative stability of TP53 autoantibodies in blood, an antibody reaction caused by a small lesion may be detectable over an extended period of time, suggesting, and confirming results by Lu et al. [21], that autoantibodies to TP53 have potential as a screening marker. While anti-TP53 may be the best-performing serum marker for breast cancer detection, its sensitivity and specificity (namely, low false-negative and false-positive rates), are limited, suggesting the need to combine it with one or several other well-performing complementary markers. More research is needed to identify such markers.

Lastly, the presence of antibodies to TP53 is weakly correlated with increased body mass. This is an unexpected finding because obesity is associated with an attenuated immune response [84] and one would therefore have expected decreased anti-TP53 with increasing BMI. Replication is needed to confirm this result; measurement of BMI is advised in studies using autoantibodies to TP53.

## Conclusions

1. Serum proteins examined and miR-135b failed to identify women with HRN or TN breast cancer.
2. TP53 autoantibodies identified women with HRN or TN breast cancer and may have potential for early detection, confirming earlier findings.
3. TP53 autoantibodies are long lasting in serum but may be affected by storage duration.

## Supporting Information

S1 Fig ROC curves of the 15 analytes. HRN breast cancer cases (43, blue) or TN breast cancer cases (28, black) vs. 87 matched controls.
(PDF)

S2 Fig Dot plot. Figure showing the values of the 15 evaluated analytes measured in 43 HRN breast cancer cases and 87 matched controls.
(PDF)

S3 Fig Anti-TP53 expression and time of storage. When adjusted for the time each serum was stored in the freezer, anti-TP53 values of the first draw (diagnostic for patients) and the second draw (post-therapy for patients) from each individual correlated (p<0.005 for cases and p = 0.029 for controls).
(PDF)

S4 Fig Correlation between anti-TP53 expression and body-mass-index (BMI). As depicted in the graph, there is a weak correlation (Kendal correlation statistic = 0.317; p = 0.005) between anti-TP53 expression and BMI.
(PDF)

S1 Table Patient Characteristics. Listing the following characteristics: Surgery Year, Time from mammogram (Mx) to Draw (weeks:days), Time Draw to Surgery (weeks:days), BI-RADS at Diagnosis (Dx), Density at Dx, Ethnicity, Death as of Dec 2014, Recurrence as of June 2014, No Ovaries at Dx, ER/PR status, HER2 status, Ducal histology, Stage, Lymph Nodes (positive / total), Metastasis, Neo-adjuvant treatment (Tx), Age at Dx, Age at Menarche, Age at Menopause, BMI, Age at first contraceptive, Current Hormone Therapy, Years of Hormone Therapy, Currently pregnant, Parity, Children, Smoking (Pack years), Drinking (g Alc / yr), Other Cancer(s).
(CSV)

S2 Table Raw data (normalized) of all 15 analytes. (CSV)

S3 Table Selection of 15 analytes and sensitivity/specificity as serum markers. Numbers are given for the full 43 HRN set and the TN subset.
(PDF)

S4 Table Bootstrap Analysis for the 43 HRN cases. (PDF)

S5 Table Bootstrap Analysis for the 28 TN cases. (PDF)

S6 Table Anti-TP53 in diagnostic (D) and post-therapy (P) draws. (PDF)

S7 Table Correlation of Patient and Disease Characteristics with Anti-TP53. (PDF)
