# Correction: Evaluating Serum Markers for Hormone Receptor-Negative Breast Cancer

**DOI:** 10.1371/journal.pone.0147561

**Published:** 2016-01-15

**Authors:** Michèl Schummer, Jason Thorpe, Maria D. Giraldez, Lindsay Bergan, Muneesh Tewari, Nicole Urban

The third author’s name is listed incorrectly. The correct name is Maria D. Giraldez. The correct citation is: Schummer M, Thorpe J, Giraldez MD, Bergan L, Tewari M, Urban N (2015) Evaluating Serum Markers for Hormone Receptor-Negative Breast Cancer. PLoS ONE 10(11): e0142911. doi:10.1371/journal.pone.0142911
