# Supplementary figures and images for: Evaluating Serum Markers for Hormone Receptor-Negative Breast Cancer
Source: PLoS One. 2015 Nov 13;10(11):e0142911. doi: 10.1371/journal.pone.0142911 (PMC4643893; doi:10.1371/journal.pone.0142911)

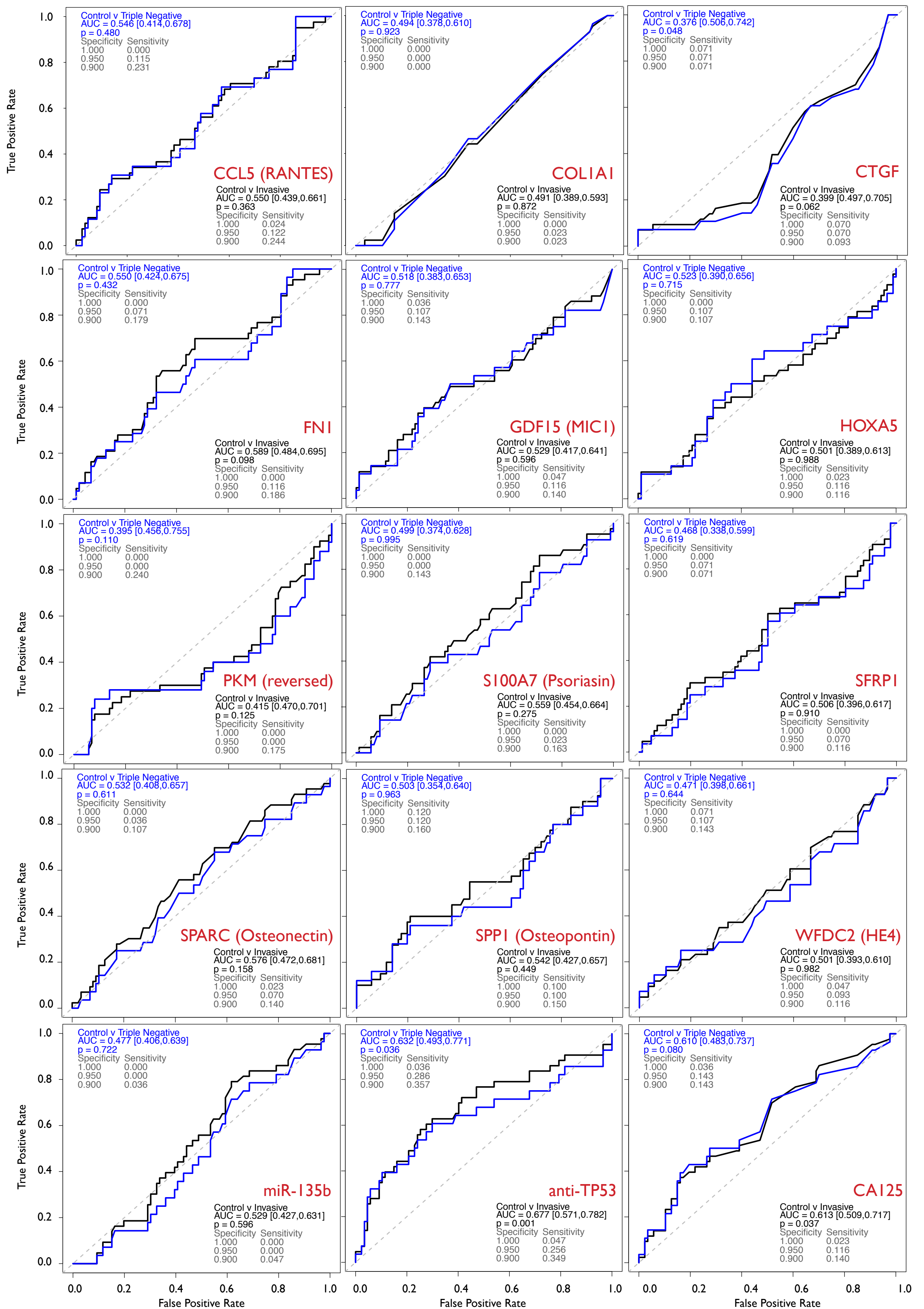

Supplement: S1 Fig — HRN breast cancer cases (43, blue) or TN breast cancer cases (28, black) vs. 87 matched controls. (PDF) [file pone.0142911.s001.pdf]
