# Supplementary material for: Evaluating Serum Markers for Hormone Receptor-Negative Breast Cancer
Source: PLoS One. 2015 Nov 13;10(11):e0142911. doi: 10.1371/journal.pone.0142911 (PMC4643893; doi:10.1371/journal.pone.0142911)
Supplement: S2 Fig — Figure showing the values of the 15 evaluated analytes measured in 43 HRN breast cancer cases and 87 matched controls. (PDF) [file pone.0142911.s002.pdf]

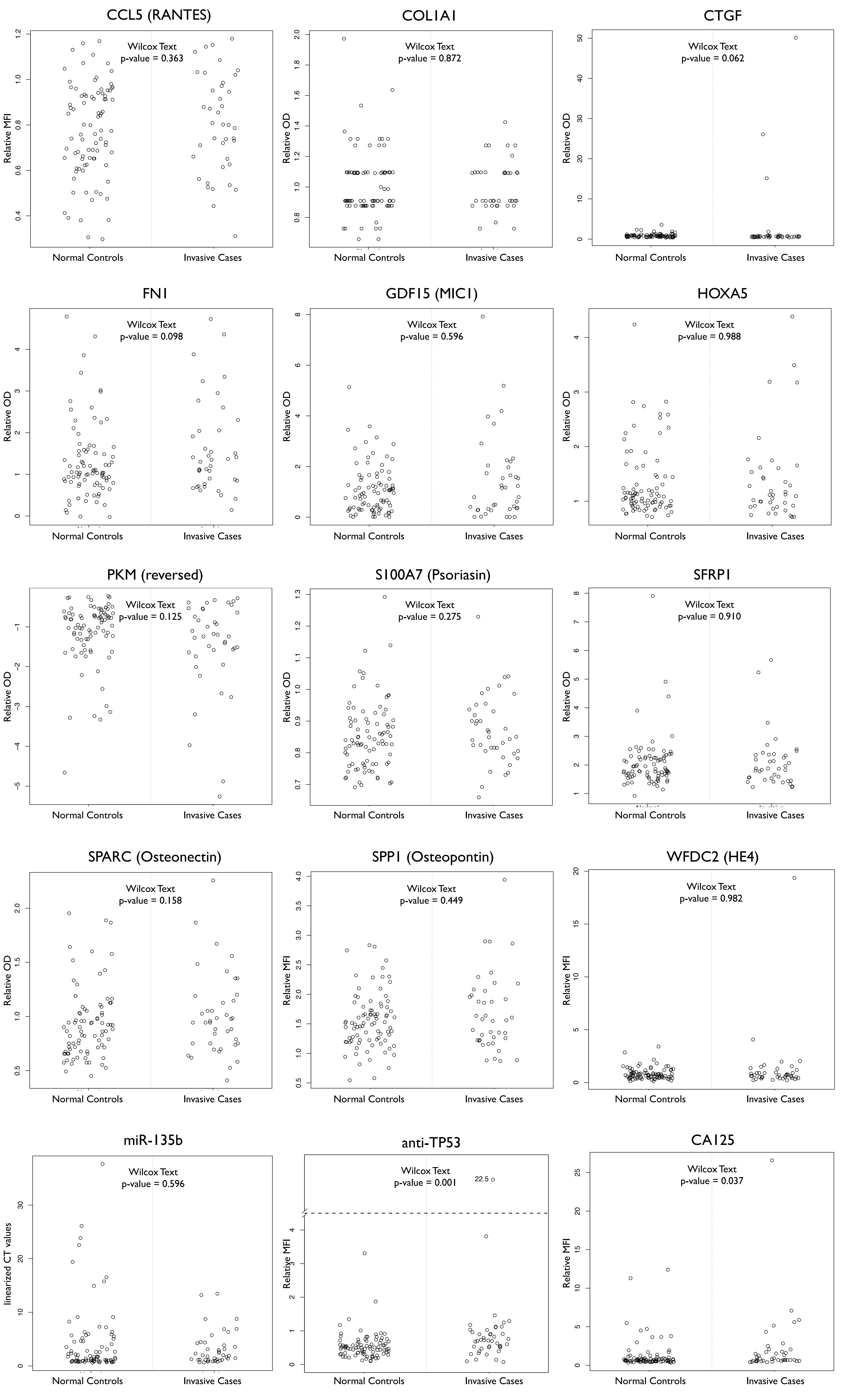
CCL5 (RANTES)
Wilcox Text
p-value = 0.363
Relative MFI
Normal Controls
Invasive Cases
COL1A1
Wilcox Text
p-value = 0.872
Relative OD
Normal Controls
Invasive Cases
CTGF
Wilcox Text
p-value = 0.062
Relative OD
Normal Controls
Invasive Cases
FN1
Wilcox Text
p-value = 0.098
Relative OD
Normal Controls
Invasive Cases
GDF15 (MIC1)
Wilcox Text
p-value = 0.596
Relative OD
Normal Controls
Invasive Cases
HOXA5
Wilcox Text
p-value = 0.988
Relative OD
Normal Controls
Invasive Cases
PKM (reversed)
Wilcox Text
p-value = 0.125
Relative OD
Normal Controls
Invasive Cases
S100A7 (Psoriasin)
Wilcox Text
p-value = 0.275
Relative OD
Normal Controls
Invasive Cases
SFRP1
Wilcox Text
p-value = 0.910
Relative OD
Normal Controls
Invasive Cases
SPARC (Osteonectin)
Wilcox Text
p-value = 0.158
Relative OD
Normal Controls
Invasive Cases
SPP1 (Osteopontin)
Wilcox Text
p-value = 0.449
Relative MFI
Normal Controls
Invasive Cases
WFDC2 (HE4)
Wilcox Text
p-value = 0.982
Relative MFI
Normal Controls
Invasive Cases
miR-135b
Wilcox Text
p-value = 0.596
linearized CT values
Normal Controls
Invasive Cases
anti-TP53
Wilcox Text
p-value = 0.001
22.5
Relative MFI
Normal Controls
Invasive Cases
CA125
Wilcox Text
p-value = 0.037
Relative MFI
Normal Controls
Invasive Cases
