# Supplementary material for: Evaluating Serum Markers for Hormone Receptor-Negative Breast Cancer
Source: PLoS One. 2015 Nov 13;10(11):e0142911. doi: 10.1371/journal.pone.0142911 (PMC4643893; doi:10.1371/journal.pone.0142911)
Supplement: S3 Fig — When adjusted for the time each serum was stored in the freezer, anti-TP53 values of the first draw (diagnostic for patients) and the second draw (post-therapy for patients) from each individual correlated (p<0.005 for cases and p = 0.029 for controls). (PDF) [file pone.0142911.s003.pdf]

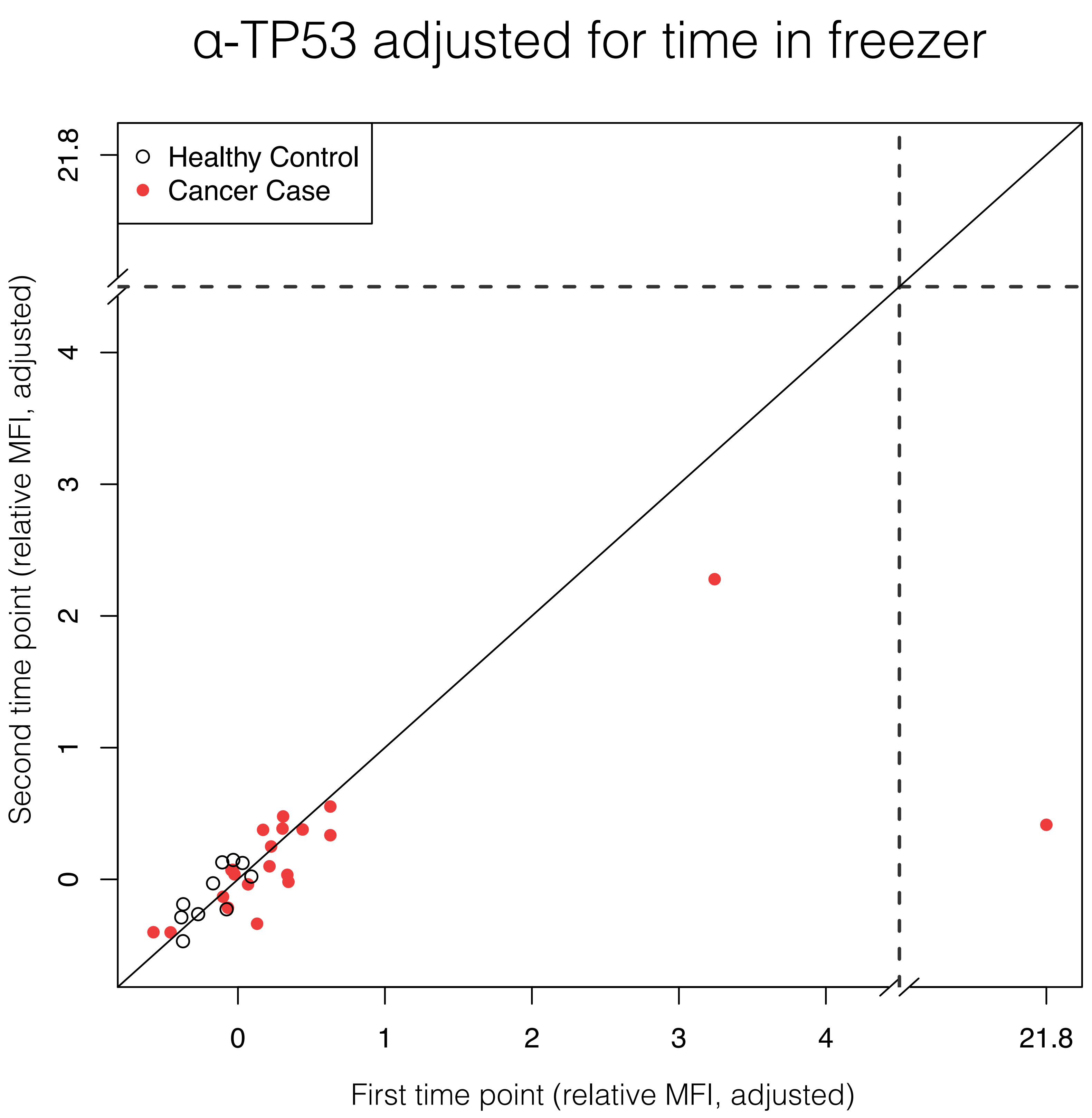

Supplemental Figure 3 – Anti-TP53 expression and time of storage. When adjusted for the time each serum was stored in the freezer, anti-TP53 values of the first draw (diagnostic for patients) and the second draw (post-therapy for patients) from each individual correlated ($p<0.005$ for cases and $p=0.029$ for controls).
