# Supplementary material for: Evaluating Serum Markers for Hormone Receptor-Negative Breast Cancer
Source: PLoS One. 2015 Nov 13;10(11):e0142911. doi: 10.1371/journal.pone.0142911 (PMC4643893; doi:10.1371/journal.pone.0142911)
Supplement: S4 Fig — As depicted in the graph, there is a weak correlation (Kendal correlation statistic = 0.317; p = 0.005) between anti-TP53 expression and BMI. (PDF) [file pone.0142911.s004.pdf]

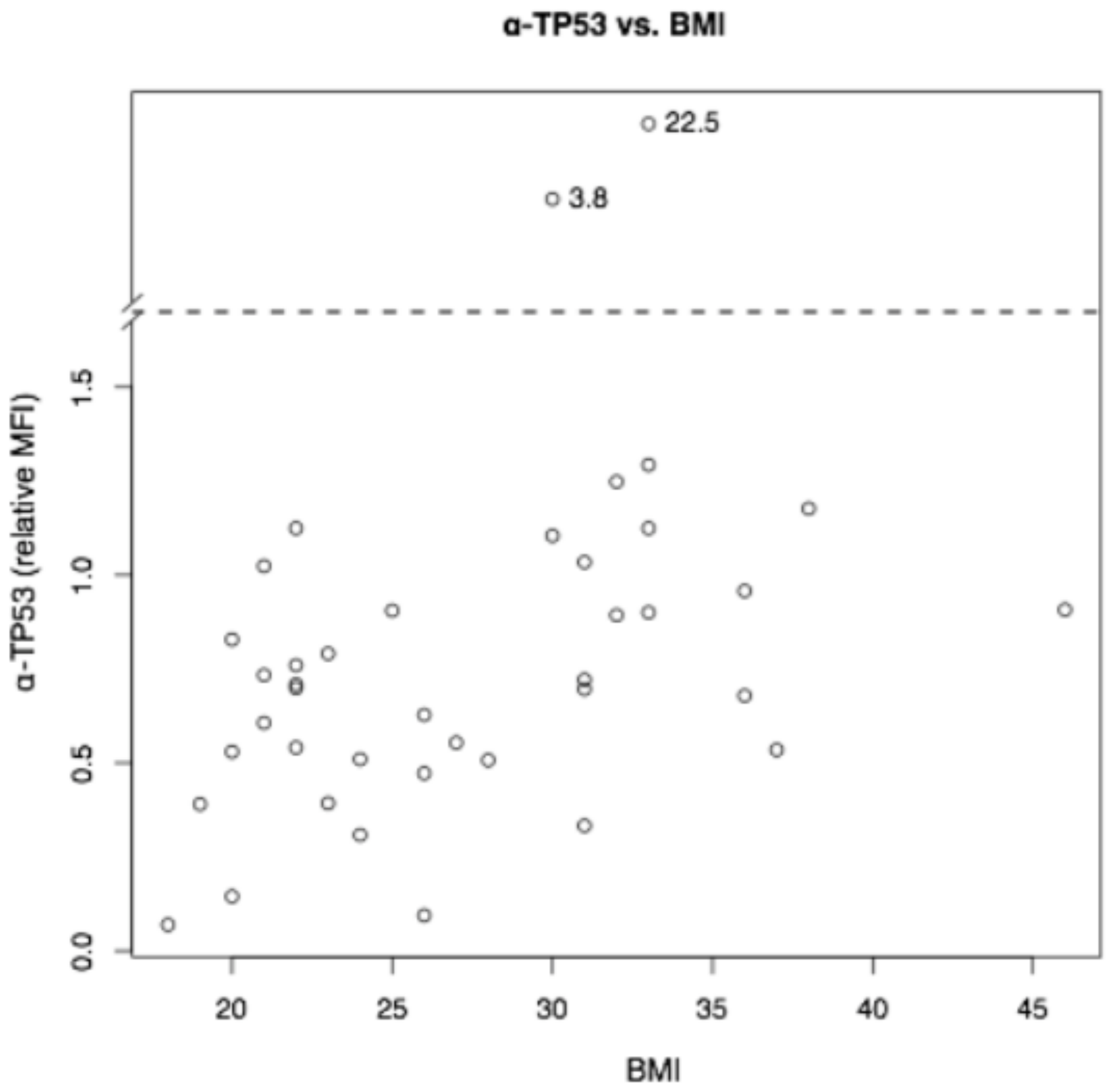

Supplemental Figure 4 – Correlation between anti-TP53 expression and body-mass-index (BMI). As depicted in the graph, there is a weak correlation (Kendal correlation statistic= 0.317; p=0.005) between anti-TP53 expression and BMI.
