# Supplementary material for: Evaluating Serum Markers for Hormone Receptor-Negative Breast Cancer
Source: PLoS One. 2015 Nov 13;10(11):e0142911. doi: 10.1371/journal.pone.0142911 (PMC4643893; doi:10.1371/journal.pone.0142911)
Supplement: S3 Table — Numbers are given for the full 43 HRN set and the TN subset. (PDF) [file pone.0142911.s007.pdf]

S3 Table – Selection of 15 analytes; Sensitivity and specificity as serum markers

		43 HRN cases and 87 controls			28 TN cases and 87 controls		
	Selection Criterion	Sensitivity at 100% specificity	Sensitivity at 95% specificity	Sensitivity at 90% specificity	Sensitivity at 100% specificity	Sensitivity at 95% specificity	Sensitivity at 90% specificity
**CA125**	1	2.3%	11.6%	14.0%	3.6%	14.3%	14.3%
**GDF15**	1	4.7%	11.6%	14.0%	3.6%	10.7%	14.3%
**PKM**	1	0.0%	0.0%	17.5%	0.0%	0.0%	24.0%
**SPARC**	1	2.3%	7.0%	14.0%	0.0%	3.6%	10.7%
**WFDC2**	1	4.7%	9.3%	11.6%	7.1%	10.7%	14.3%
**COL1A1**	2	0.0%	2.3%	2.3%	0.0%	0.0%	0.0%
**CTGF**	2	7.0%	7.0%	9.3%	7.1%	7.1%	7.1%
**FN1**	2	0.0%	11.6%	18.6%	0.0%	7.1%	17.9%
**S100A7**	2	0.0%	2.3%	16.3%	0.0%	0.0%	14.3%
**SPP1**	2	10.0%	10.0%	15.0%	12.0%	12.0%	16.0%
**CCL5**	3	2.4%	12.2%	24.4%	0.0%	11.5%	23.1%
**miR-135b**	3	0.0%	0.0%	4.7%	0.0%	0.0%	3.6%
**Anti-TP53**	3	4.7%	25.6%	34.9%	3.6%	28.6%	35.7%
**HOXA5**	4	2.3%	11.6%	11.6%	0.0%	10.7%	10.7%
**SFRP1**	4	0.0%	7.0%	11.6%	0.0%	7.1%	7.1%

Data from 43 cases and 87 controls. Marker selection criteria: (1) serum marker potential in high-grade serous ovarian cancer and involvement in breast cancer; (2) transcript involved in breast cancer; (3) association with poor breast cancer outcome; (4) protein decreased in breast cancer tissue compared to normal breast. Changes in the TN subset relative to the full HRN set are indicated with color shading (green=better, red=worse).
