# Supplementary material for: Evaluating Serum Markers for Hormone Receptor-Negative Breast Cancer
Source: PLoS One. 2015 Nov 13;10(11):e0142911. doi: 10.1371/journal.pone.0142911 (PMC4643893; doi:10.1371/journal.pone.0142911)
Supplement: S4 Table — (PDF) [file pone.0142911.s008.pdf]

S4 Table – Bootstrap Analysis for the 43 HRN cases

Analyte Panel	Type of Statistic	Observed Statistics		Bootstrap Analyses (10,000 iterations)		
		Anti-TP53 Alone	Analyte Panel	Statistics	Percentiles	P-values
				mean (sd)	estimate [95% CI]	(2 sided)
Anti-TP53 with FN1 and CTGF (selected using AIC)	AUC	0.66	0.65	0.67 (0.035)	0.726 [0.717, 0.735]	0.548
	Sensitivity at 95% Specificity	27.5%	27.5%	0.20 (0.071)	0.068 [0.063, 0.073]	0.136
	Sensitivity at 90% Specificity	37.5%	27.5%	0.28 (0.081)	0.474 [0.464, 0.484]	0.948
Anti-TP53 with SPP1, FN1, CCL5 and GDF15 (selected based on sensitivity at 95% and 90% specificity)	AUC	0.66	0.63	0.65 (0.030)	0.722 [0.713, 0.730]	0.557
	Sensitivity at 95% Specificity	27.5%	25%	0.16 (0.060)	0.056 [0.051, 0.060]	0.111
	Sensitivity at 90% Specificity	37.5%	27.5%	0.24 (0.068)	0.243 [0.234, 0.251]	0.486

The panels were fit and all statistics were calculated using the 43 cases and 87 controls for which data from all 5 markers were available. AIC: Akaike's Information Criterion. AUC: Area under the curve.
