# Supplementary material for: Evaluating Serum Markers for Hormone Receptor-Negative Breast Cancer
Source: PLoS One. 2015 Nov 13;10(11):e0142911. doi: 10.1371/journal.pone.0142911 (PMC4643893; doi:10.1371/journal.pone.0142911)
Supplement: S5 Table — (PDF) [file pone.0142911.s009.pdf]

S5 Table – Bootstrap Analysis for the 28 TN cases

Analyte Panel	Type of Statistic	Observed Statistics		Bootstrap Analyses (10,000 iterations)		
		Anti-TP53 Alone	Analyte Panel	Statistics mean (sd)	Percentiles estimate [95% CI]	P-values (2 sided)
Anti-TP53 with FN1, CTGF and WFDC2 (Selected using the AIC criterion)	AUC	0.61	0.65	0.71 (0.055)	0.828 [0.820, 0.835]	0.345
	Sensitivity at 95% Specificity	30.8%	30.8%	0.23 (0.093)	0.142 [0.135, 0.149]	0.283
	Sensitivity at 90% Specificity	38.5%	34.6%	0.33 (0.1)	0.381 [0.372, 0.391]	0.762
Anti-TP53 with FN1, CA125, and WFDC2 (selected based on sensitivity at 95% and 90% specificity)	AUC	0.61	0.63	0.67 (0.039)	0.872 [0.865, 0.878]	0.256
	Sensitivity at 95% Specificity	30.8%	26.9%	0.18 (0.076)	0.091 [0.086, 0.097]	0.183
	Sensitivity at 90% Specificity	38.5%	30.8%	0.27 (0.084)	0.247 [0.239, 0.256]	0.495

The panels were fit and all statistics were calculated using the 28 triple-negative (TN) cases and 87 controls for which data from all 5 markers were available. AIC: Akaike's Information Criterion. AUC: Area under the curve.
