# Supplementary material for: Evaluating Serum Markers for Hormone Receptor-Negative Breast Cancer
Source: PLoS One. 2015 Nov 13;10(11):e0142911. doi: 10.1371/journal.pone.0142911 (PMC4643893; doi:10.1371/journal.pone.0142911)
Supplement: S6 Table — (PDF) [file pone.0142911.s010.pdf]

S6 Table – Anti-TP53 in diagnostic (D) and post-therapy (P) draws

Patient #	Kind	Interval D to P	Anti-TP53 (D)	Anti-TP53 (P)	P / D
**1**	Cancer	76w 4d	0.91	0.50	0.55
**3**	Cancer	103w 0d	0.07	0.11	1.52
**4**	Cancer	48w 4d	1.25	1.11	0.89
**5**	Cancer	81w 4d	22.45	0.92	0.04
**10**	Cancer	56w 0d	0.15	0.13	0.87
**11**	Cancer	57w 2d	1.03	0.90	0.87
**12**	Cancer	118w 1d	0.90	0.91	1.01
**14**	Cancer	109w 0d	0.55	0.52	0.93
**16**	Cancer	25w 5d	0.54	0.62	1.15
**17**	Cancer	53w 4d	0.51	0.29	0.57
**20**	Cancer	60w 1d	0.70	0.15	0.22
**21**	Cancer	51w 1d	3.81	2.78	0.73
**25**	Cancer	48w 1d	0.53	0.53	1.00
**27**	Cancer	61w 3d	0.72	0.85	1.17
**29**	Cancer	48w 3d	0.89	0.47	0.52
**30**	Cancer	80w 3d	1.18	0.78	0.66
**31**	Cancer	52w 1d	0.76	0.58	0.76
**32**	Cancer	70w 1d	0.68	0.48	0.71
**34**	Cancer	52w 0d	0.73	0.69	0.94
**35**	Cancer	53w 5d	0.39	0.29	0.74
**36**	Cancer	54w 6d	0.79	0.80	1.01
**44**	Control	107w 3d	0.27	0.03	0.11
**52**	Control	114w 4d	0.17	0.20	1.19
**53**	Control	83w 4d	0.57	0.55	0.97
**57**	Control	59w 2d	0.30	0.22	0.76
**77**	Control	53w 6d	0.38	0.44	1.18
**90**	Control	118w 2d	0.22	0.16	0.73
**99**	Control	118w 4d	0.51	0.53	1.04
**104**	Control	107w 2d	0.47	0.56	1.20
**124**	Control	172w 6d	0.54	0.16	0.30
**131**	Control	16w 6d	0.65	0.56	0.86

Comparison of two blood draws from the same cancer patient or healthy control. For cancers, D is the diagnostic draw and P is the post-therapeutic draw. For the controls, D and P are two blood draws with intervals similar to those for the 21 cancer patients.
