# Supplementary material for: Evaluating Serum Markers for Hormone Receptor-Negative Breast Cancer
Source: PLoS One. 2015 Nov 13;10(11):e0142911. doi: 10.1371/journal.pone.0142911 (PMC4643893; doi:10.1371/journal.pone.0142911)
Supplement: S7 Table — (PDF) [file pone.0142911.s011.pdf]

S7 Table – Correlation of Patient and Disease Characteristics with Anti-TP53

Covariate	Kendall Correlation Statistic	p-Value
Days from Draw to Surgery	0.057	0.603
BI-RADS	-0.041	0.738
Breast Density	-0.210	0.104
Death	-0.050	0.695
Recurrence	-0.005	0.968
No ovaries	0.040	0.755
Triple-negative cancer	-0.097	0.445
Stage	-0.048	0.678
Positive Lymph Nodes	-0.128	0.327
Metastases	0.138	0.290
Neoadjuvant Treatment	-0.139	0.277
Age at Diagnosis	-0.054	0.615
Age at Menarche	-0.027	0.826
Age at Menopause	0.080	0.511
Tumor size	0.029	0.796
BMI	0.317	0.005
Parity	0.066	0.559
Children	0.015	0.894
Smoking (Pack years)	0.058	0.625
Alcohol intake (g Alc per yr)	-0.076	0.502
Previous Cancer	-0.150	0.227
